# Monitoring vegetation dynamics and carbon stock density in miombo woodlands

**DOI:** 10.1186/1750-0680-8-11

**Published:** 2013-11-09

**Authors:** Natasha S Ribeiro, Céu N Matos, Isabel R Moura, Robert A Washington-Allen, Ana I Ribeiro

**Affiliations:** 1 Department of Forest Engineering, Eduardo Mondlane University, P.O. Box 257, Maputo, Mozambique; 2 Agrobiodiversity Unit, Tropical Research Institute, Quinta do Marquês, Oeiras 2784-505, Portugal; 3 Department of Geography, University of Tennessee, Burchfiel Geography Building, 1000 Phillip Fulmer Way, Knoxville TN37996-0925, USA; 4 Department of Ecosystem Science & Management, Texas A&M University, TAMU 2138, College Station, TX 77843-2138, USA

**Keywords:** REDD+, Miombo woodlands, Carbon stock density, Conservation areas

## Abstract

**Background:**

The United Nation’s Program for Reducing Emissions from Deforestation and Forest Degradation (REDD+) aims to reduce the 20% contribution to global emissions of greenhouse gases from the forest sector, offering a financial value of the carbon stored in forests as an incentive for local communities. The pre-requisite for the setup of a participatory REDD + Program is the monitoring, reporting and verification (MRV) of baseline carbon stocks and their changes over time. In this study, we investigated miombo woodland’s dynamics in terms of composition, structure and biomass over a 4-year period (2005–2009), and the Carbon Stock Density (CSD) for the year 2009. The study was conducted in the Niassa National Reserve (NNR) in northern Mozambique, which is the 14th largest protected area in the world.

**Results:**

Mean tree density distributed across 79 species increased slightly between 2005 and 2009, respectively, from 548 to 587 trees ha^-1^. *Julbernardia globiflora* (Benth.) was the most important species in this area [importance value index (IVI_2005_*=* 61 and IVI_2009_ = 54)]. The woodlands presented an inverted J-shaped diametric curve, with 69% of the individuals representing the young cohort. Woody biomass had a net increase of 3 Mg ha^-1^ with the highest growth observed in *Dyplorhynchus condilocarpon* (Müll.Arg.) Pichon (0.54 Mg ha^-1^). *J. globiflora* had a net decrease in biomass of 0.09 Mg ha^-1^. Total CSD density was estimated at ca. 67 MgC ha^-1^ ± 24.85 with soils (average 34.72 ± 17.93 MgC ha^-1^) and woody vegetation (average 29.8 MgC ha^-1^ ± 13.07) representing the major carbon pools. The results point to a relatively stable ecosystem, but they call for the need to refocus management activities.

**Conclusions:**

The miombo woodlands in NNR are representative of the woodlands in the eco-region in terms of vegetation structure and composition. They experienced net increase in woody biomass, a considerable recruitment level and low mortality. According to our results, NNR may present good potential for carbon sequestration especially in soils and woody biomass, representing an important potential carbon sink. However, further investigations are needed in order to address the contribution of this area to MRV REDD + initiatives.

## Background

The United Nation’s Program for Reduction in Emissions from Deforestation and Forest Degradation in Developing Countries (REDD+) aims to enhance the livelihoods of subsistence economies by facilitating the development of voluntary markets and international agreements to credit communities for afforestation and conservation of carbon activities [1,2]. The Intergovernmental Panel on Climate Change (IPCC) has provided guidelines on methodologies to assess carbon stocks [2-4]. Although remote sensing is a key tool to assess carbon stocks over large areas [1], operational field methods provide higher levels of accuracy and confidence [3,4].

Tropical savannas and woodlands are a major component of the world’s vegetation, covering 1/6 of the land surface and over 1/2 of the African continent. They account for about 30% of the primary production of all terrestrial vegetation, playing a crucial role in energy, water and carbon balance [5-10].

Miombo woodlands cover two-thirds of the Sudan-Zambezian phytoregion (ca. 2.4 million km^2^) representing an important plant diversity center that extends over seven countries: Angola, the Democratic Republic of Congo, Malawi, Mozambique, Tanzania, Zambia and Zimbabwe [11]. Miombo is characterized by the overwhelming dominance of *Brachystegia*, *Julbernardia* and *Isoberlina* tree species, but its overall plant diversity is as high as 8,500 species including other tree, grass, herb and shrub species [12].

The structure and composition of miombo are strongly determined by their woody component, particularly by large trees, which play a key role in ecosystem function [13-15], primarily in nutrient cycling that accounts for a great deal of the carbon pool [16]. This component is in turn constrained by a combination of climate, disturbances [e.g. drought, fire, and herbivory primarily by elephants (*Loxodonta africana* Blumenbach)] and human activities [17-20]. There is increasing concern that the loss of mature trees in landscapes subjected to deforestation and degradation, as well as intense fires may result in the transformation of the woodlands into scrub or grasslands [8,19,21-23] with the associated loss of biodiversity and biomass and thus an increase in carbon emissions.

Given the importance of the miombo woodlands as a reservoir of above- and below-ground carbon, it presents potential for implementation of REDD + policies towards environmental sustainability and socio-economic development [6-9]. However, research on carbon dynamics is still incipient (e.g. [8,10]) and it needs to be evaluated more systematically. Within this context, the purpose of this study was to conduct an IPCC Tier II [4] investigation of the change in vegetation dynamics in the miombo woodlands from the Niassa National Reserve (NNR) in order to 1) explore the woodland’s dynamics in terms of structure, composition and biomass between 2005 and 2009; and 2) estimate the ecosystem carbon stock density (CSD) for the year 2009. NNR is one of the most pristine conservation areas of miombo woodlands in southern Africa and is probably a large repository of carbon, thus representing a key conservation area for MRV REDD + initiatives [24-26].

## Results

### Woodland dynamics in 4 years: structure, composition and biomass

**Figure 1:**
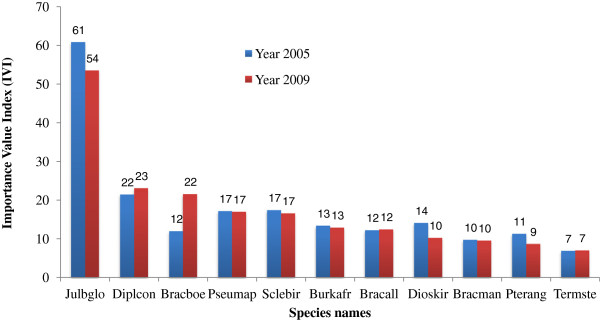
**Importance Value Index (IVI) by species in 2005 and 2009.** Legend: Julbglo (*Julbernardia globiflora*); Diplcon (*Diplorhynchus condilocarpon*); Bracboe (*Brachystegia boehmii*); Pseumap (*Pseudolachnostylis maprouneifolia*); Sclebir (*Sclerocarya birrea*); Burkafr (*Burkea africana*); Bracall (*B. allenii*); Dioskir (*Diospyros kirkii*); Bracman (*B. manga*); Pterang (*Pterocarpus angolensis*); Termste (*Terminalia stenostachya*).

A total of 1933 individuals (548 trees ha^-1^) belonging to 79 tree species were recorded in 2005 and a total of 1990 individuals (587 trees ha^-1^) were recorded in 2009. In both years, the top 11 ecologically important tree species, expressed by the importance value index (IVI), were: *Julbernardia globiflora* (IVI_2005_ = 61 and IVI_2009_ = 54)*, Diplorhynchus condylocarpon* (IVI_2005_ = 22 and IVI_2009_ = 23), *Brachystegia boehmii* Taub. (IVI_2005_ = 12 and IVI_2009_ = 22)*, Pseudolachnostylis maprouneifolia* Pax.var. *maprouneifolia* (IVI = 17 in both years), *Sclerocarya birrea* (A. Rich.) Hochst. (IVI = 17 in both years), *Burkea africana* Hook. (IVI = 13 in both years), *Brachystegia allenii* Burtt Davy & Hutch. (IVI = 12 in both years), *Diospyros kirkii* Hiern (IVI_2005_ = 14 and IVI_2009_ = 10), *Brachystegia manga* De Wild. (IVI = 10 in both years), *Pterocarpus angolensis* DC. (IVI_2005_ = 11 and IVI_2009_ = 9) and *Terminalia stenostachya* Engl. & Diels (IVI = 7 in both years) (Figure 1). Altogether, these species accounted for 65% of the total IVI in both years. Other ecologically important species found in this study were *Combretum hereroense* Schinz (IVI = 5 in both years), *Catunaregam spinosa* (Thunb) Tirveng (IVI = 5 in both years), *Combretum zeyheri* Sond. (IVI_2005_ = 5 and IVI_2009_ = 4), among others. At the natural regeneration level we found 55 species that were not present in the adult stage and 48 species also present in the adult classes.

Horizontal structure measured as the diametric distribution was typical of tropical and sub-tropical forests with an inversed J-shape diametric curve. In both years 69% of the individuals were represented in the young cohort (diameter at breast height – dbh: 5-15-cm), while only 0.55% of individuals were represented in the adult class (> 45-cm of dbh). Vertical structure indicated two tree strata with a mean dominant height of 8-m (varying from 5 to 22-m). Nearly 80% of the trees were established in the sub-canopy stratum (between 7 and 10.5-m) and only 11% were represented in the upperstorey (height above 19-m). The lower stratum was basically composed of grass and herbaceous species.

The total woody biomass estimated in 2009 was 59 Mg ha^-1^ ± 26.15 (ranging from 15 to 165 Mg ha^-1^), which represents an increase in biomass of 3 Mg ha^-1^ (or 5%) in 4 years. Seventy six percent of the 25 most abundant species presented a net growth of biomass ranging from 0.01 to 0.54 Mg ha^-1^, while 24% had a negative net growth (Table 1 for the top 10 species). The highest growth was observed for *D. condylocarpon* (0.54 Mg ha^-1^), followed by *B. allenii* (0.40 Mg ha^-1^), *P. maprouneifolia* (0.29 Mg ha^-1^) and *B. africana* (0.25 Mg ha^-1^). Other ecologically important species such as *B. boehmii* (0.11 Mg ha^-1^) and *T. stenostachya* (0.14 Mg ha^-1^) experienced smaller net biomass growth, while *J. globiflora* experienced a negative net biomass of -0.09 Mg ha^1^.

**Table 1:** Biomass stocks, mortality and net biomass changes for the 10 most dynamic tree species in Niassa National Reserve

**Species name**	**Biomass 2005 (Mg ha**^**-1**^**) ± Stdev**	**Biomass 2009 (Mg ha**^**-1**^**) ± Stdev**	**Mortality (Mg ha**^**-1**^**) ± Stdev**	**Net biomass changes (Mg ha**^**-1**^**)**
*Diplorhynchus condilocarpon*	1.82 ± 0.8	2.38 ± 0.85	0.02 ± 0.01	0.54
*Brachystegia allenii*	3.26 ± 1.02	3.70 ± 1.03	0.04 ± 0.01	0.40
*Burkea africana*	3.08 ± 1.01	3.33 ± 1.00	0.00	0.25
*Pseudolachnostylis maprouneifolia*	2.42 ± 0.85	2.73 ± 0.80	0.02 ± 0.01	0.29
*Terminalia stenostachya*	0.63 ± 0.3	0.78 ± 0.31	0.01 ± 0.005	0.14
*Brachystegia boehmii*	5.83 ± 1.2	5.96 ± 1.12	0.02 ± 0.01	0.11
*Dyospiros kirkii*	1.30 ± 0.95	1.39 ± 0.90	0.00	0.09
*Combretum hereroense*	0.48 ± 0.02	0.53 ± 0.01	0.02 ± 0.01	0.03
*Julbernardia globiflora*	1.44 ± 0.75	1.57 ± 0.73	0.22 ± 0.015	-0.09
*Pterocarpus angolensis*	2.18 ± 1.20	1.88 ± 1.19	0.06 ± 0.01	-0.36

Biomass of recruiting individuals (those entering the diametric class of 5 cm in 2009) varied between 0 and 0.28 Mg ha^-1^. *B. boehmii* presented the most prominent recruitment (0.28 Mg ha^-1^), followed by *D. condylocarpon* (0.23 Mg ha^-1^)*, Brachystegia spiciformis* Benth. (0.19 Mg ha^-1^)*, P. maprouneifolia* (0.13 Mg ha^-1^) and *B. allenii* (0.13 Mg ha^-1^). The rest of the species presented less than 0.1 Mg ha^-1^ of ingrowth.

Mortality was in general very low for most tree species, varying between 0 and 0.22 Mg ha^-1^. *J. globiflora* presented the highest mortality of 0.22 Mg ha^-1^, followed by *P. angolensis* and *B. spiciformis* (both with ca. 0.055 Mg ha^-1^).

### Ecosystem carbon stock density

The estimated Carbon Stock Density (CDS) for the year 2009 was used in this study as the reference data, given that it represents the most updated and complete (above and belowground) estimation as compared to the data from 2005. The total CSD estimated for this area was 67 MgC ha^-1^ (Stdev ± 24.85). The major contributors to the CSD were soils and woody vegetation. The former represented ca. 52% (average 34.72; Stdev ± 17.93 MgC ha^-1^) of the total CSD, ranging from 8 to 89.8 MgC ha^-1^ and the latter corresponded to ca. 45% of the total CSD (average 29.88; Stdev ± 13.07 MgC ha^-1^), varying from 10 to 79.7 MgC ha^-1^ (Table 2). The error estimation was 7.14, indicating 90% level of precision. *J. globiflora* had the greatest contribution with 9 MgC ha^-1^, followed by *P. maprouneifolia*, *B. africana*, *B. boehmii* and *B. manga* with contributions ranging from 1 to 3 MgC ha^-1^.

**Table 2:** Carbon Stock Density (CSD) of miombo woodlands in Niassa National Reserve

**Ecosystem Compartment (EC)**	**Biomass (Mg ha¯**^**1**^**)**	**Stdev**	**Carbon Sotck Density (CSD) (MgC ha¯**^**1**^**)**	**Stdev**	**SE**	**% of total CSD**
**Trees**	59.00	26.15	29.88	13.07	0.26	45
**Dead Trees**	0.12	0.37	0.06	0.19	0.003	-
**Grasses**	4.05	1.78	2.03	0.89	0.018	3
**Litter**	0.12	0.06	0.06	0.03	0.001	-
**Herbaceous**	0.04	0.03	0.02	0.01	0.0002	-
**Soil**	-	-	34.72	17.93	0.36	52
**Total**	63.33	28.00	66.77	24.85	0.28	100

## Discussion

This study presents the dynamics of miombo woodlands over a 4-year growth period and ecosystem carbon stock density (CSD) for the year 2009 in 50 permanent sample plots across NNR. It is unlikely that the analyses reveal all the existing variations within the area given its large extension (42,000 km^2^), the short period of time of this study (4-years), the low representative sampling effort and the limited accessibility of the area. However, the results are in sequence of previous studies conducted by the authors and collaborators [8,19,24,27], which together show consistent ecological patterns.

### Woodlands dynamics over the 4-year study period (2005–2009)

Structure and species composition of the woodlands reveal a typical miombo ecosystem, dominated by *Julbernardia globiflora, Brachystegia boehmii, Pseudolachnostylis maprouneifolia, Diplorhynchus condylocarpon* and *Burkea africana*. Tree density and woody biomass presented a concomitant increase, respectively 57 ind. ha^-1^ (i.e. 3%) and 3 Mg. ha^-1^ (i.e. 5%) in 4 years. At the species level, both parameters were also concurrent, i.e. biomass accumulation accompanied species ecological expression. For example, *D. condilocarpon* was the most prominent species in biomass growth and had an increase in IVI from 22 to 23, while *J. globiflora* experienced negative net biomass growth (due to low biomass recruitment and high mortality) and had a decrease in IVI from 61 to 54. Our results indicate that the woodlands in NNR were dynamic and presented a trend that is within the range found elsewhere in the miombo eco-region (380 to 1,400 trees ha^-1^ and more than 50 species) [19,28-37].

The horizontal and vertical structures were stable over the 4-year study period. The woodland had a healthy size-class distribution with a higher number of juvenile trees (5–15 cm dbh) than adults (> 45 dbh). This structure is commonly found across the miombo region and is usually an expression of the dominant canopy of Caesalpinoideae trees [12]. However, the dominant height found in this study (8 m) is lower than expected for dry miombo (10–15 m) [38,39]. This may be explained by a combination of factors such as poor soils, climatic conditions and disturbances [8,12,19,27]. It is well known that miombo soils are in general nutrient-poor and NNR is not an exception. For example [40], notes that in drier places (with shallow soils or with less soil moisture storage capacity) of the reserve the woodland is shorter and more open.

Disturbances, especially fires and elephants, are also important factors in shaping the ecosystem structure and composition, creating a woodland of lower density and stature at some places of NNR [8,12,19,27,41]. Elephants uproot and de-branch large trees promoting the grass component, which feeds late dry-season fires. The latter shapes the ecosystem by removing saplings and young individuals of specific tree species.

The fact that the biomass and ecological expression of *J. globiflora* has decreased from 2005 to 2009 may also be an indication of the influence of fires, since it is known that this species is fire-sensitive and tends to decline under regular burnings [12]. Furthermore, *J. globiflora* along with others species in NNR are influenced by human land management practices. For example, local communities use some tree species, such as *J. globiflora*, for several purposes including bark-based beehives, timber production, food or medical applications (N. Ribeiro, unpublished results).

### Carbon stock density (CSD)

Estimation of CSD is a basic step in carbon accounting and consideration of land use options and strategies to promote carbon sequestration. Benchmark sites are vital as they allow determination of deviations under different land uses. This is particularly important in the miombo eco-region in which the diversity of soils, climate, hydrology and disturbances return highly variable CSDs making a comparison among sites not always possible [9,42]. In fact, CSD values in NNR ranged from 10 to 80 MgC ha^-1^ among sampling plots, which may be attri buted to its large extension of 42,000 Km^2^ and associated variation in environmental conditions including fire frequency and elephant density [12].

Overall the mean CSD of ca. 67 MgC ha^-1^ (Stdev ± 26.15) and a woody vegetation CSD of ca. 30 Mg C ha^-1^ (Sdev ± 13.07; 45% of the total CSD in NNR) were slightly higher when compared with other studies in the miombo eco-region (Table 3) and comparable to the estimations of Ribeiro et al. [8] conducted in the same area (35 MgC ha^-1^). The total precision of 11% indicates that the estimations in this study were within the precision range (10-20%) required for this type of analysis [43,44]. *J. globiflora* is a major contributor to the ecosystem’s CSD representing 30% of woody CSD and 13% of the total CSD. As discussed previously in this paper, the species dominates the area but its ecological value and biomass decreased in 4 years (IVI decreased from 61 to 54) likely as a result of a combined result of fires and human land management practices. This points to the need to refocus management activities in NNR to prioritize human land management practices, especially fires. Otherwise major changes may be imposed on this ecosystem, which in turn may lead to a decrease in CSD especially of dominant species such as *J. globiflora*.

**Table 3:** Comparative results of carbon stock density with other similar studies across the miombo eco-region

**Ecosystem compartment**	**This study (Mg C ha**^**-1**^**)**	**Other studies (Mg C ha**^**-1**^**)**	**Localization**	**Reference**
**Soil**	34.72 ± 17.93	57.90	Gorongosa, Mozambique	[9]
		31.04	Dombe, Manica, Mozambique	[45]
**Trees**	29.88 ± 13.07	19 ± 8	Gorongosa, Mozambique	[9]
		13.17 - 32.10	Beira Corridor, Mozambique	[46]
		20.88	Niassa, Mozambique	Sitoe, Unpublished data
		26.48	Dombe, Manica, Mozambique	[45]
		35.00	Niassa National Reserve	[8]
**Grasses**	2.03 ± 0.89	1.2	Niassa, Mozambique	Sitoe, Unpublished data
		0.65	Dombe, Manica, Mozambique	[45]
**Litter**	0.06 ± 0.03	0.8	Niassa, Mozambique	Sitoe, Unpublished data
		3.0	Dombe, Manica, Mozambique	[45]
**Dead Trees**	0.06 ± 0.19	-	-	-
**Herbaceous**	0.02 ± 0.01	0.55 ± 0.02	Eastern Arc Mountains, Tanzania	[42]
**Total carbon**	10.13-79.69	13 - 30	Eastern Arc Mountains, Tanzania	[42]

CSD in the first 30 cm of soil represents the major carbon pool in this area as expected for the miombo ecosystem [9,45,47]. Soil carbon in miombo woodlands is not widely studied and reported. However, a few studies indicate that the conversion of miombo woodlands to short-duration croplands is a major cause of carbon release from soils in the region [43,45,48]. As stated previously [27] fires are a major ecological factor in the reserve but their influence on soil CSD is a matter of further investigation and questions of how to improve soil CSD by managing fire may be of interest.

Although our CSD estimates are not representative of the entire NNR (for the reasons described above), the added value of this study in the context of REDD + lies in the fact that it gives an overview of CSD and carbon pools in this area. The results revealed that at the current level of management (more focused on wildlife and less on vegetation, fires and human activities) NNR can still be considered an important spot for carbon markets. Further estimations would be necessary in order to contribute to the national MRV REDD + initiatives. Those should include among others, the development of local allometric equations, and systematic and extensive carbon estimations by increasing the sampling effort and calibration of high-resolution remote sensing data. What constitutes the appropriate management approach in terms of REDD + will have to be determined in consultation with local communities, NNR managers as well as forest authorities. One major limitation would be the limited capacity of local communities in establishing partnerships. However, the experiences of benefit sharing through devolution of 20% of the conservation fees appear to be a good starting point to involve communities in forest management.

## Conclusions

Our study revealed that the miombo woodlands in NNR are a good representation of miombo woodlands in the region. Species composition and structure followed the trend in the eco-region with dominance of typical miombo tree species. In 4 years the ecosystem experienced a slight increase in woody biomass, a considerable recruitment level and low mortality. However, one of the most important species – *Julbernardia globiflora* - showed a decrease in IVI and in biomass. This may indicate that the species is under pressure given its relative importance for local livelihoods and low tolerance to fires.

NNR presents good potential for carbon sequestration especially in soils and woody biomass. Even though our results may not be representative of the entire area, they represent a useful benchmark against which other estimates can be compared. Further and more accurate carbon estimates need to be performed to accurately account for carbon in the area, before a decision is made on engaging in REDD + initiatives.

Overall, the authors recommend that management activities and practices in NNR focus on fire management and human land-use practices. These would include among other things defining a concrete community-based fire management program and engage local communities in sustainable forest management activities.

## Methods

### Study site

**Figure 2:**
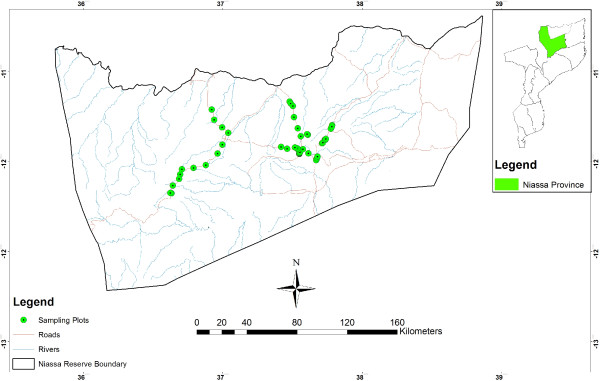
Location of Niassa National Reserve.

This study was conducted in Niassa National Reserve (NNR), a 42,000 km^2^ conservation area that is located in northern Mozambique between 12°38′48.67″S and 11°27′05.83″S and 36°25′21.16″E and 38°30′23.74″E [8,19,49,50] (Figure 2).

The climate is tropical sub-humid, with a mean annual precipitation (MAP) of 900-mm that increases from the east (800-mm) to the west (1,200-mm) and a mean annual temperature (MAT) of 25°C that ranges from 20 to 26°C during the dry season (May – October). The wet season (November – April) has a MAP of 900-mm and a MAT of 30°C. NNR has a gently undulating landscape on a plateau with elevations ranging from 300 to 600-m above-sea-level (asl).

According to White [51], NNR is classified as “drier Zambezian miombo woodland” with intrusions of east African coastal elements along the Rovuma and Lugenda rivers. Ribeiro et al. [8] determined that 82% of the reserve is miombo woodland. Low and medium-density woodlands occupy the lowland areas. High-density woodlands are located on the slopes of the Mecula and Jao inselbergs in the central portion of the reserve, and along the mainstreams in the form of riverine forests. This study was conducted in the lowland miombo woodlands.

The woodlands in NNR are floristically poorer than the wet miombo and are dominated by the presence of *Brachystegia spiciformis*, *Brachystegia boehmii* and *Julbernardia globiflora*[12]. Other ecologically important species in the area are: *Pseudolachnostylis maprouneifolia, Pterocarpus angolensis* and *Diplorhynchus condylocarpon. Combretum* spp. in the east and *Uapaca* spp*.* in the west are also present in NNR [19]. The canopy is generally less than 15-m in height and the trees are deciduous for a month or more during the dry season.

### Data acquisition

Field sampling was conducted in 2 years (2005 and 2009) according to the methods reported in [19]. Fifty 30-m diameter circular plots were established in July 2004 and measured in 2005 and 2009. Each plot was geographically referenced using a Geographic Positioning System (GPS) unit (Garmin III +, Garmin Inc.). Individual species were identified according to [52] and [53]. Diameter-at-breast height (dbh), height, and ingrowth were measured for all trees with dbh > 5-cm in July 2005 and July 2009. Ingrowth was considered as all the individuals entering the 5-cm dbh size class by 2009. Every individual of the ingrowth cohort was measured for dbh and height in 2009. Mortality was measured in each plot as the number of individuals found dead during the study period. Natural regeneration was measured by the number of individuals with dbh < 5-cm and height between 0.3 and 1-m. Grass biomass was measured in 2005 and 2009 along two transects within every plot, using the Disc Pasture Meter (DPM) developed by Bransby and Tainton [54] for African savannas and calibrated for the miombo woodlands in southern Africa. Litter and herbaceous biomass were estimated by collecting 15 samples of each component in every plot, weighting them for green weight (kg) and drying until constant weight in a Labcon, EFDO kiln at 105°C.

Twelve soil samples per plot at 0 to 30 cm depth were analyzed for carbon and bulk density using the Walkley-Black method [55].

### Data analysis

To achieve a nearly complete description of species composition and distribution this study used the importance value index - IVI [56]. IVI was calculated by adding up three characteristics of a particular species: relative frequency (how often a species occurs in the plots), relative abundance (density of plants) and relative dominance [density of stock expressed as basal area, calculated from Eq. (2)]. Thus, the IVI is an indicator of ecosystem importance and is frequently used as a quantifier for vegetation studies. Stock density was calculated as:

(1) $$\mathrm{BA}=\left(\pi /40000\right)\times \left(\mathrm{db}h^{2}/a\right)$$

Where BA = basal area (m^2^ ha^-1^), dbh = diameter at breast height and *a* = area of a plot (= 0.071 ha).

Tree species biomass dynamics between 2005 and 2009 was explored for the 25 tree species that presented 20 or more individuals as they correspond to representative populations in the sampled area [57]. Population biomass dynamics was explained in terms of mortality, growth, recruitment (or ingrowth) in biomass and richness of natural regeneration (in number of species) during the 4-year period.

Annual woody biomass was estimated for 2005 and 2009 using an allometric equation developed by Mugasha and Chamshama [58] for miombo woodlands with similar edaphic and climatic conditions^a^:

(2) $$\mathrm{WB}=b0\times \left(\mathrm{db}h^{b1}\right)$$

Where, WB = woody biomass (kg tree^-1^), dbh in cm, b_1_ = 0.0625 and b_0_ = 2.553 corresponding to the regression coefficients.

Biomass of trees that died during the study period of 4 years was calculated using Eq. (3) from Marzoli [59]:

(3) $$\mathrm{DB}=\text{vol}\times \left(\mathrm{MD}/A\right)$$

Where: *DB* = dead biomass (ton ha-^1^), *vol* = volume of the dead tree (m^3^), *MD* = mean timber density for miombo species (0.65 for miombo woodlands of central and northern Mozambique) and *A* = sampling area (3.55 ha).

Net biomass changes for each species were calculated using Eq. (4):

(4) $$\text{Net}\;\text{biomass}\;\text{change}=\left(\mathrm{WB}2009-\mathrm{WB}2005\right)-\mathrm{DB}$$

Where: WB2009 = woody biomass for the year 2009 (ton ha-^1^), WB2005 = woody biomass for the year 2005 ((ton ha-^1^) and DB = dead biomass (ton ha-^1^).

CSD for the miombo ecosystem in NNR was considered in this study as the carbon in the aboveground vegetation (woody, grass, herbaceous and litter) and superficial soil (30 cm depth). The biomass of all the vegetation components (live trees, dead vegetation, grasses, herbaceous and litter) was converted to carbon using the 0.5 conversion factor [60]. Soil carbon was estimated using Eq. (5) from Pearson et al. [44]:

(5) SC=BD×D×C×100

Where: *SC* = soil carbon content (ton ha-^1^), *BD* = bulk density (g m-^3^), *D* = depth of soil sample collection (30 cm) and *C* =% carbon content estimated in the laboratory. Carbon estimation precision (*P*) was estimated using Eq. (6) from Pearson et al. [44]:

(6) P=E/μ

Where: E error of estimation calculated as  E = √ [(t^2^) × ((N × s)^2^/n) - (N × s)^2^)], *μ* = mean carbon density (ton ha^-1^), *t* = *“t”* for 95% confidence interval (= 2), *N* = proportion of sampled area in relation to the total areas of NNR (8.07 × 10^-7^), *n* = number of sampling plots (50), *s* = standard deviation of the mean.

## Endnote

^a^Eq. (1) was developed in the Kitulangalo forest in Morogoro, Tanzania under similar edaphic–climatic conditions as Niassa National Reserve. The site is dominated by open miombo woodland. The climate is tropical and sub humid within 700 and 1000 mm of mean annual precipitation and a mean annual temperature of 24.3°C [58].

## Abbreviations

AGBC: Aboveground biomass and carbon; CSD: Carbon stock density; Dbh: Diameter-at-breast height; DPM: Disc pasture meter; GPS: Geographic positioning system; IPCC: Intergovernmental panel on climate change; IVI: Importance value index; MAP: Mean annual precipitation; MAT: Mean annual temperature; MRV: Monitoring, reporting and verification; NNR: Niassa national reserve; REDD+: Reducing emissions from deforestation and forest degradation.

## Competing interests

The authors declare they have no competing interests.

## Author’ contributions

NSR conceived the study, coordinated data collection, analysis and interpretation and the draft of the manuscript. CNM participated in data collection and analysis. IRM has been involved in data analysis, drafting the manuscript and revising it critically. RAWA has made substantial contributions to acquisition and interpretation of data and helped to draft the manuscript. AIR has made substantial contributions to data analysis and interpretation and co-coordinated the drafting of the manuscript. All authors read and approved the final manuscript.

## Authors’ information

NSR: Associate Professor at the Faculty of Agronomy and Forest Engineering since 2013 has been conducting research in the Niassa National Reserve since 2004. Her main research areas are carbon balance and effects of disturbances on miombo structure and composition. NSR is the coordinator of the Miombo Network of Southern Africa.

CNM: Masters student in Agrarian Development with emphasis in forest and fauna resources management. CNM is also working at the Ministry of tourism in Mozambique.

IRM: Senior Researcher at the Tropical Botanical Garden, Tropical Research Institute (IICT), Portugal. Main research focus, biodiversity conservation. Since 2007 she has been collaborating with NSR in the study of biodiversity, fire and carbon dynamics in the Niassa National Reserve.

RAWA: Assistant Professor at the Department of Geography, the University of Tennessee. Research Interests: applied science problems to provide solutions to questions concerning possible land degradation, climate change, invasive species, conservation biology, and ecological restoration.

AIR: Senior Researcher with Habilitation and Deputy Director of Biotrop – Environment, Agriculture and Development Center, Tropical Research Institute (IICT), Portugal. Main research focus: genetic resources, biodiversity and plant-environment interactions. Since 2007 she has been collaborating with NSR in the study of biodiversity, fire and carbon dynamics in the Niassa National Reserve.
